# Disparity in the regulation and prevention of water versus sodium imbalance in heat-stress nephropathy: a phylogenetic perspective

**DOI:** 10.1093/ckj/sfag151

**Published:** 2026-05-21

**Authors:** Michele Cirillo, Carmine Zoccali, Carlo Garofalo, Silvio Borrelli, Chiara Ruotolo, Federica Marzano, Roberto Minutolo, Luca De Nicola, Giuseppe Conte

**Affiliations:** Nephrology Unit, University of Campania Luigi Vanvitelli, Naples, Italy; Associazione Ipertensione Nefrologia Trapianto Renale, c/o Nefrologia, Grande Ospedale Metropolitano, Reggio Calabria, Italy; Nephrology Unit, University of Campania Luigi Vanvitelli, Naples, Italy; Nephrology Unit, University of Campania Luigi Vanvitelli, Naples, Italy; Nephrology Unit, University of Campania Luigi Vanvitelli, Naples, Italy; Nephrology Unit, University of Campania Luigi Vanvitelli, Naples, Italy; Nephrology Unit, University of Campania Luigi Vanvitelli, Naples, Italy; Nephrology Unit, University of Campania Luigi Vanvitelli, Naples, Italy; Nephrology Unit, University of Campania Luigi Vanvitelli, Naples, Italy

**Keywords:** chronic kidney disease, dehydration, evolutionary physiology, heat-stress nephropathy, sodium balance

## Abstract

The progressive rise in global temperature has led to an increase in heat-related illness and has brought heat-stress nephropathy (HSN) to the forefront as an emerging cause of both acute kidney injury (AKI) and chronic kidney disease (CKD). HSN is characterized by tubulo-interstitial damage and linked to disturbances in water and sodium homeostasis. Observational studies indicate that young, otherwise healthy workers exposed to heat are less likely to develop AKI when hypovolaemia due to sweating is corrected with sodium chloride-containing solutions rather than water alone. This narrative review first summarizes the clinical spectrum, epidemiology, pathophysiology, and prevention of HSN in the context of climate change and the broader epidemic of heat-associated CKD described in several tropical and temperate regions. Then, it examines, in a phylogenetic perspective, the evolution of the two main regulatory systems governing body fluid homeostasis: the antidiuretic hormone (ADH)–thirst axis (water balance) and the renin–angiotensin–aldosterone system (RAAS)–salt appetite axis (sodium balance). In aquatic environments, large external osmotic gradients drove early renal adaptations primarily focused on water handling. Approximately 600 million years ago, ADH-like nonapeptides emerged, enabling tight regulation of plasma osmolality. With the transition to terrestrial life, the development of long loops of Henle, a hypertonic renal medulla, and exquisitely sensitive thirst mechanisms allowed mammals to conserve water very efficiently. By contrast, the RAAS system, central to sodium conservation and effective circulating volume, appeared later (around 400 million years ago) and remains slower and less sensitive, with no behavioural drive equivalent to thirst. The mineralocorticoid receptor is present in fish, but its specific ligand aldosterone first appears in terrestrial vertebrates. The net result is a phylogenetically more refined defence of water than of sodium. We propose that this evolutionary asymmetry underlies the particular renal vulnerability observed in HSN, in which inadequate sodium replacement and suboptimal control of volaemia may predispose to ischaemic tubular injury. Understanding these evolutionary roots may help explaining why, in the era of global warming, the prevention of HSN requires not only water but also appropriate salt replacement and targeted protection of vulnerable populations.

## Graphical Abstract

**Graphical Abstract:**
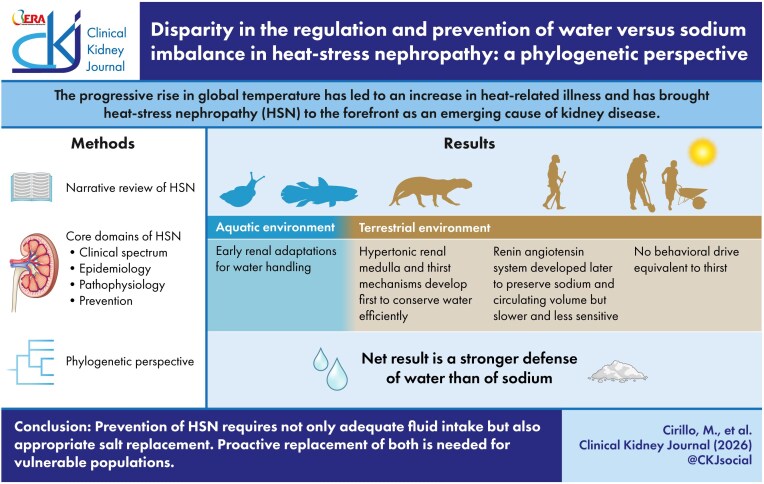

## INTRODUCTION

Over the past 30 years, the global mean temperature has increased by almost 1°C, in line with a broader warming trend that has exceeded 1.5°C since the beginning of the twentieth century and has accelerated in the last decade [1]. Depending on future greenhouse gas emissions, median temperature increase of about 1.4°C above pre-industrial levels is expected by 2030 and up to 3°C or more by 2100 [1]. As temperatures rise, more people are exposed to frequent, intense heat waves, with important consequences for kidney health in both tropical and temperate climates [2, 3]. A recent study has examined the association between heat exposure and incidence and prevalence of kidney disease at a macro county-level scale across USA, linking ambient temperatures and socioeconomic and geographic factors to renal outcomes, thereby advancing understanding of environmental determinants and strategies to mitigate disparities in affected communities [4]. Heat-related kidney disease was recently described as a ‘rampant epidemic’ in regions increasingly affected by extreme heat [5].

In several rural areas of Central America, India, and Sri Lanka, a distinctive form of chronic kidney disease (CKD) has been described in predominantly young male agricultural workers, often in the absence of diabetes and long-standing hypertension [2, 3, 6–10]. Similar patterns are now reported in other hot environments, including parts of China and the Mediterranean basin [9, 11–13]. Affected individuals usually perform intense manual labour outdoors under high temperatures and present with reduced glomerular filtration rate (GFR), minimal or no proteinuria, and histological features of chronic tubulo-interstitial nephritis with associated glomerulosclerosis [9, 10]. In many settings, disease clusters in communities where heat exposure, heavy workloads, and limited access to safe water or effective occupational protection coexist, although nonagricultural workers and adolescents can also be affected [2, 3, 9, 10]. This emerging picture has focused attention on the kidney as a vulnerable target of climate change and chronic heat stress.

There is increasing recognition that disturbances in water and sodium balance may be linked to heat-related kidney injury. Recurrent episodes of dehydration and extracellular volume depletion due to sweating, combined with cutaneous vasodilation and redistribution of blood flow, reduce renal perfusion and oxygen delivery and predispose to repeated episodes of acute kidney injury (AKI) [14–18]. Workers often ingest large volumes of water to compensate for fluid losses without proportionate sodium replacement. This mismatch between strong drives to restore water balance and weaker mechanisms for sodium conservation may contribute to heat-stress nephropathy (HSN) and may help explaining why tubulo-interstitial injury is so prominent.

### Methods

This article is a narrative, hypothesis–generating review. We identified relevant publications through nonsystematic searches of PubMed and Google Scholar using combinations of the terms ‘heat stress’, ‘chronic kidney disease’, ‘heat–stress nephropathy’, ‘dehydration’, ‘hyperosmolarity’, ‘sodium balance’, ‘salt appetite’, ‘RAAS’, and ‘evolutionary physiology’. Additional references were obtained from the bibliographies of key articles and prior reviews. We prioritized epidemiological studies from heat–exposed populations, experimental and physiological studies on water and sodium regulation, and comparative-phylogenetic work on osmoregulation and thermoregulation. Since the goal was conceptual integration rather than quantitative synthesis, we did not perform a formal systematic review, risk–of–bias assessment, or meta–analysis, and the evidence summarized here should be interpreted accordingly.

### Scope of this review

This narrative review focuses on HSN as a clinical and pathophysiological entity within the broader spectrum of heat-related kidney diseases and climate-sensitive CKD. Its first objective is to summarize current knowledge on the clinical spectrum, epidemiology, mechanisms, and prevention of HSN, with particular attention to disturbances of water and sodium balance. The second objective is to examine, from a phylogenetic perspective, the evolution of renal and hormonal systems that regulate water and sodium [antidiuretic hormone (ADH)–thirst axis and the renin–angiotensin–aldosterone system (RAAS)–salt appetite axis] and to show how their different evolutionary trajectories may shape present-day response to heat and volume stress. Third objective is to integrate these clinical and evolutionary insights into a framework for understanding renal vulnerability under heat stress and to derive practical implications for clinical care, occupational health, and public policy. We do not discuss specific aetiological controversies; instead, we use available data to frame HSN as a multifactorial condition in which heat and fluid-electrolyte disturbances may play a central role.

## HSN: CLINICAL SPECTRUM, PATHOPHYSIOLOGY, AND PREVENTION

### Definition and clinical picture

HSN is a heat-related kidney disorder that has gained attention in parallel with rising global temperatures. It has been most extensively described in tropical regions such as Mesoamerica, Sri Lanka, and parts of India; however, more recent data show that it also affects populations in temperate climates, including Europe, the USA, and China [2, 3, 9, 11–13]. Clinically, HSN spans a spectrum from transient, often subclinical AKI to progressive CKD and, in some cases, end-stage kidney disease (ESKD).

In many cohorts, HSN presents predominantly as Kidney Disease: Improving Global Outcomes stage 1 or 2 AKI [19]; nevertheless, cases of advanced CKD and dialysis-dependent ESKD in relatively young, previously healthy individuals have also been reported [9, 10, 18, 20]. During periods of intense physical exertion in hot environments, renal blood flow progressively declines; in combination with hypovolaemia due to sweating, this reduction can exceed 50% [14, 15]. Cross-shift studies in agricultural workers have documented AKI incidences between 11% and 25% during a single work shift under heat stress [16–18]. Even short-term exposure to high temperatures and heavy labour is associated with a significantly increased risk of kidney injury [16–18].

In Central American sugarcane workers, rises in serum creatinine and tubular biomarkers correlate with high ambient temperatures, heavy workloads, and markers of volume depletion [16–18, 21]. A longitudinal study in Guatemalan sugarcane workers showed that repeated daily episodes of mild AKI across the harvest season were associated with cumulative estimated glomerular filtration rate (eGFR) loss [22]. In a referral hospital in El Salvador, 67% of incident ESKD cases were not attributable to diabetes, hypertension, or primary glomerulonephritis, but occurred predominantly in young male farmers working in high-temperature regions [20]. Beyond these hotspots, a Taiwanese longitudinal study found that individuals with heat-related illnesses had a higher risk of subsequent CKD compared with matched controls [23]. Moreover, a *post hoc* analysis of the DAPA-CKD trial showed that ambient heat exposure was associated with more rapid GFR decline in patients with pre-existing CKD [24].

Table 1 summarizes the typical clinical picture of HSN, including demographic features, laboratory findings, and histological patterns.

**Table 1:** Clinical picture of HSN.

Domain	Evidence	Key findings
Early kidney injury markers	Urinary biomarkers of tubular damage	Urinary biomarkers of renal tubular injury become detectable before an increase in serum creatinine, indicating subclinical kidney damage.
Heat-related AKI	AKI prevalence in healthy individuals exposed to heat stress	Heat-related AKI occurs in 12.3% of subjects after a single workday and in 25% after eight consecutive workdays.
Heat stress and CKD progression	*Post hoc* analysis of DAPA-CKD trial	Heat stress is associated with a more rapid and severe progression of CKD in patients with established CKD.
HSN and long-term outcomes	HSN in individuals without traditional CKD risk factors	HSN may develop in subjects without advanced age, diabetes, or hypertension, and in the absence of urinary abnormalities, and can be followed by CKD and an increased incidence of ESKD.
Renal histopathology	Kidney biopsy findings	Absence of vascular lesions, autoimmune antibodies, and inflammatory cellular infiltrates; presence of tubular atrophy and interstitial fibrosis.

**Table 2:** Prevention of HSN by administration of electrolyte solutions.

Authors	Type of study	Findings
Chicas *et al*. 2022	Randomized Controlled Trial	In 30 workers exposed to high heat, drinking electrolyte solutions did not induce any case of AKI, while drinking water without electrolytes induced 23% of AKI.
Butler-Dawson *et al*. 2019	Observational	Sugarcane workers with higher electrolyte solution intake were less likely to develop AKI.
Laws *et al*. 2015	Observational	For every additional electrolyte solution packet consumed by cane cutters, mean late harvest eGFR was 6.1 ml/min/1.73 m^2^ higher.
Krisher *et al*. 2020	Observational	In heat-stressed workers with increased electrolyte intake, cases of rhabdomyolysis decreased from 44% to 8%, while renal function remained stable.

### Early biomarkers and histopathology

In acute heat-stress settings, early kidney injury may be detected by sensitive biomarkers before changes in serum creatinine become evident [21]. Exercise under heat stress is associated with increased urinary excretion of tubular injury markers including β2-microglobulin, cystatin C, neutrophil gelatinase-associated lipocalin, osteopontin, and uromodulin (Table 1) [21, 25]. The risk of AKI increases with rising core body temperature, particularly above 38°C [17, 25].

Histological studies in communities exposed to hot climates—especially but not exclusively agricultural workers—reveal a relatively homogeneous pattern. Biopsies typically lack significant vascular lesions, autoantibodies, or dense cellular inflammatory infiltrates [9, 10]. Although we highlight an evolutionary asymmetry between water and sodium regulation, this framework operates within a broader spectrum of environmental and social determinants that may equal or surpass its causal importance in HSN. In many affected regions, chronic exposure to nephrotoxins such as agrochemicals, contaminated drinking water, heavy metals, and over–the–counter or prescribed nonsteroidal anti-inflammatory drugs (NSAIDs) can directly induce tubular and interstitial injury and may also potentiate damage from recurrent heat stress and dehydration. Infectious exposures, including recurrent gastrointestinal infections with volume depletion, endemic viral or bacterial pathogens, and subclinical urinary tract infections, can further accelerate loss of nephron mass and promote low–grade inflammation. Superimposed on these biological factors, powerful socioeconomic drivers can be hypothesized: intense manual labour at high temperatures, piece–rate or informal employment structures that discourage breaks, limited access to shade and cooling, constrained opportunities for safe hydration during work shifts, and poor access to preventive and curative healthcare. These conditions not only increase the frequency and severity of heat and osmotic stress, but also modify patterns of fluid and sodium intake and the timing of rehydration, thereby shaping how any underlying evolutionary asymmetry in water and salt defence is expressed clinically. In this sense, evolutionary biology provides a useful lens for understanding susceptibility, but the realized risk of HSN emerges from the interaction of these intrinsic regulatory traits with exogenous toxic, infectious, and socioeconomic exposures.

### HSN as a three–way homeostatic trade–off

**Figure 1:**
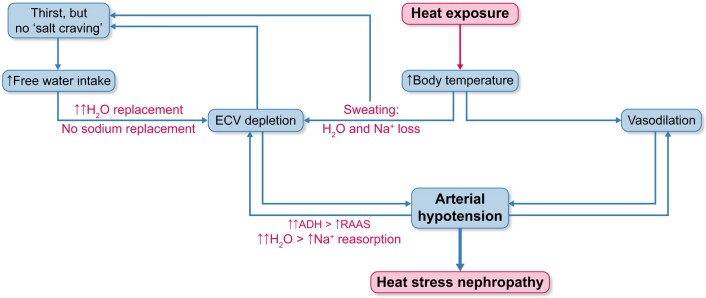
HSN Pathogenesis

HSN can be viewed as the outcome of a three–way balance between water homeostasis, sodium/volume regulation, and thermoregulation. From an evolutionary perspective, the maintenance of core body temperature within a narrow range is a primary survival goal, because even modest elevations in body temperature can impair cellular function and threaten vital organ integrity [26, 27]. To protect core temperature, the organism ‘accepts’ substantial water and sodium losses through sweating, thereby enhancing evaporative cooling at the expense of extracellular volume and osmotic stability. In this framework, water and salt conservation systems are continually trading off against the thermoregulatory imperative, especially in individuals exposed to high environmental heat and heavy physical work. At the renal level, repeated or sustained heat stress can induce tubular ATP depletion and mitochondrial dysfunction, promoting a pattern of injury that shares features with acute interstitial nephritis rather than classic ischaemic acute tubular necrosis (ATN) [28]. Local inflammatory pathways, endothelial activation, and altered microvascular tone further compromise oxygen delivery to vulnerable medullary segments [29]. These processes are amplified when hyperosmolality and volume contraction develop: hemoconcentration and reduced perfusion intensify medullary hypoxia, while hypertonic stress augments inflammatory signalling and cellular injury. Thus, HSN emerges not exclusively from disordered water or sodium balance, but also from the dynamic interaction of water conservation, volume regulation, and temperature control, with thermoregulatory demands often taking precedence and making the kidney particularly susceptible to structural damage under conditions of recurrent heat exposure (Fig. 1).

### Prevention: the role of sodium-containing solutions

Preventive strategies for HSN must be interpreted with caution, as the current evidence base is heterogeneous, largely observational, and context dependent. While several studies suggest that combined water and sodium replacement may mitigate acute kidney stress in some high–heat occupational settings, these findings come from diverse protocols, small samples, and differing baseline diets, workloads, and comorbidities [22, 30–32]. Accordingly, we now view sodium–containing solutions not as a universal recommendation, but as one potential component of tailored strategies for selected high–risk groups (e.g. heavily sweating, otherwise healthy workers with high salt losses), ideally guided by local climatic conditions, work intensity, and typical dietary sodium intake. At the same time, increased sodium intake may pose important risks for individuals with hypertension, established CKD, heart failure, or salt–sensitive blood pressure, and for populations already consuming high–sodium diets. In such groups, a focus on adequate water intake, work–rest cycles, cooling measures, and occupational and environmental modifications may be safer than routine sodium supplementation. Overall, preventive approaches should be individualized, integrated with broader occupational and public–health interventions, and implemented with explicit recognition of residual uncertainty. Well–designed prospective and interventional studies are needed before endorsing widespread public–health policies that systematically promote sodium–containing solutions for heat–exposed populations. Table 2 summarizes preventive studies which show that electrolyte solutions may reduce the risk of AKI and rhabdomyolysis in heat-exposed workers compared to water alone.

Recurrent heat exposure combined with intense physical exertion may lead to dehydration and extracellular volume depletion, thereby contributing to AKI and CKD. Because salt appetite is weaker and less immediate than thirst, replacement of sodium losses generally requires active behavioural or clinical interventions. Thirst, triggered by small increases in plasma osmolality, powerfully promotes water intake but does not guarantee restoration of effective circulating volume. This imbalance between water and sodium replacement may be a driver of HSN (Fig. 1) [33, 34].

To understand why our physiological defences are so asymmetric with respect to water and sodium, it is useful to revisit the evolutionary history of renal and hormonal regulation.

## THE ‘INSIDE SEA’: EARLY EVOLUTION OF WATER REGULATION

A plausible starting point for the evolution of water and sodium regulation is the aquatic life of our primordial ancestors. The electrolyte composition of amniotic fluid, derived from maternal extracellular fluid, closely resembles that of the primordial oceans, although at somewhat lower concentrations (Table 3) [35, 36]. Amniotic fluid thus mirrors both the maternal milieu intérieur and, more distantly, early marine conditions, reflecting a deep evolutionary link to the origin of life in water.

**Table 3:** Electrolyte concentrations of the major inorganic ions in primordial ocean and amniotic fluid.

	Primordial ocean (mmol/l)	Amniotic fluid (mmol/l)
Sodium	470–490	120–140
Chloride	540–560	90–110
Potassium	9–10	3.8–4.5
Calcium	10–11	1.5–2.0

**Figure 2:**
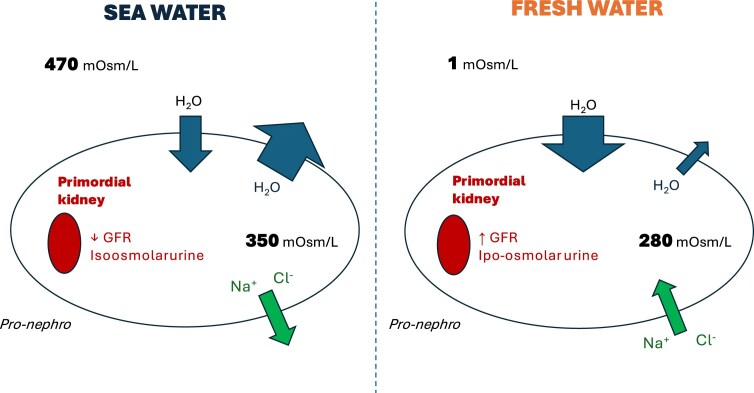
Osmoregulation in Fish: Seawater vs. Freshwater

Ocean salinity has progressively increased over geological time. By contrast, the plasma of saltwater fish has remained hypo-osmolar relative to seawater, predisposing these organisms to osmotic water loss (Fig. 2) [36, 37]. To counteract this, saltwater fish drink seawater in volumes up to 25% of their body weight per day [36, 37]. Part of the ingested sodium and chloride is excreted via gill epithelia, while renal filtration is limited. Urine is roughly iso-osmolar to plasma and the capacity to concentrate urine is minimal; plasma osmolality is therefore relatively high (∼350 mOsm/l) in marine teleosts (Fig. 2) [36, 37].

**Figure 3:**
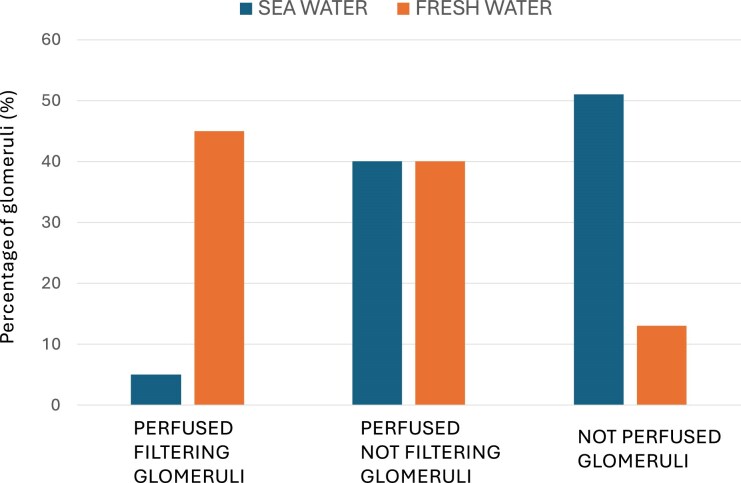
Glomerular intermittency in seawater vs. freshwater

In freshwater environments, the situation is reversed. Freshwater fish are hyperosmolar relative to their environment, so water enters the body by osmosis. Excess water must be excreted, and these species produce moderately hypotonic urine. Unlike in marine fish, where many glomeruli are functionally quiescent, freshwater fish recruit all glomeruli into active filtration to eliminate excess water, a phenomenon known as ‘glomerular intermittency’ (Fig. 3) [36, 38]. This intermittency appears to be modulated by opposite effects of angiotensin II on GFR in saltwater and freshwater fish, as shown in trout [39]. Plasma osmolality in freshwater fish is lower (∼280 mOsm/l), consistent with relative hyperhydration [36].

These adaptations illustrate that large external osmotic gradients in seawater versus freshwater generated opposing water fluxes and likely drove early renal specializations aimed primarily at water balance rather than sodium balance [36, 37, 40].

## ADAPTATION TO DRY LAND: THIRST, ADH, AND MEDULLARY HYPERTONICITY

### Thirst and behavioural regulation of water intake

The transition from aquatic to terrestrial life posed new challenges, particularly the need to seek water proactively to replace losses due to evaporation and urinary excretion. In terrestrial vertebrates, including mammals, the thirst centre resides in hypothalamic osmoreceptive neurons, located near ADH-secreting neurons and integrated into limbic circuits [41].

Experimental evidence suggests that a rudimentary thirst mechanism was already present in early terrestrial vertebrates. In toads, angiotensin II induces thirst-driven water uptake through the ventral skin (‘cutaneous drinking’). In amphibious fish, ‘anticipatory’ thirst refers to oropharyngeal signals–such as dryness–that trigger drinking before measurable deficits in total body water develop [42, 43]. This mechanism likely evolved to maintain water films over gills and later facilitated terrestrial survival by promoting rapid water intake when it became available [42, 43].

In humans, thirst is the principal defence against hyperosmolality. It is stimulated by a rise in plasma osmolality of only 1%–2% (Table 4), and, when intact, can compensate large urinary water losses, as in diabetes insipidus [44, 45]. During terrestrialization, thirst evolved into a sensitive regulator of water balance, in contrast to the weaker drive to ingest salt.

**Table 4:** Characteristics of regulatory systems of water and sodium.

	ADH	Aldosterone
Onset	600 million years ago	400 million years ago
Stimulus	1%–2% increase of plasma osmolarity	8%–10% of plasma volume loss
Mechanism of action	Rapid expression of aquaporins via cAMP	Slow antinatriuretic action via transcriptional pathways
Main defence mechanism	Thirst Central and local sensors responsive to 1%–2% increase of plasma osmolarity Active in healthy subjects and in patients with diabetes insipidus	Salt craving Undetected sensory centres Low prevalence in healthy subjects and in patients with Addison’s disease

### ADH, aquaporins, and the hypertonic renal medulla

ADH (vasopressin) is a nonapeptide highly conserved throughout vertebrate evolution. Its evolutionary precursor, vasotocin, is present in nonmammalian vertebrates and some invertebrates [46, 47]. The identification of a vasopressin-related precursor (preproconopressin) in the mollusc *Lymnaea stagnalis* indicates that the vasopressin/oxytocin superfamily already existed in a common ancestor of vertebrates and invertebrates about 600 million years ago (Table 4) [47].

Vasopressin likely played an early role in water regulation. In amphibians, organs such as the kidney, urinary bladder, and skin reabsorb water in response to vasotocin/vasopressin via aquaporin channels [46]. Although amphibians lack loops of Henle and cannot produce highly concentrated urine, they can still modulate renal water reabsorption to some extent (Table 5).

**Table 5:** Progressive improvement in the regulation of plasma osmolarity over time.

• Vasotocin, the ancestral neurohypophyseal hormone, involved in the control of the primitive hydro-osmotic cell
• In Amphibians, the nephron, the bladder, and the skin (‘cutaneous drinking’) respond to vasotocin by increasing their permeability
• Onset of thirst in terrestrial vertebrates, including Amphibians
• From glomerular intermittency to the rise of GFR and tubular reabsorption in terrestrial organisms
• In metanephric kidney, the hypertonicity of the renal medulla and the development of the long loop of Henle represent adaptive evolutions from a nonefficient water-retaining system

With the emergence of amniotes and mammals, new renal adaptations allowed vertebrates to become less dependent on external water. Long loops of Henle arranged with vasa recta created a countercurrent system capable of generating a steep osmotic gradient from cortex to papilla (Table 5) [48]. During dehydration, ADH secretion is stimulated by a 1%–2% rise in plasma osmolality, similar to the thirst threshold [42]. Under maximal ADH stimulation, the human kidney can concentrate urine up to ∼1200 mOsm/l, while some desert rodents reach even higher values, reflecting exceptionally long loops of Henle [36].

The ADH–aquaporin system, together with a hypertonic renal medulla, thus constitutes an efficient mechanism for conserving water. It emerged early and was progressively refined during the conquest of land. The result is a robust, rapid, and sensitive defence of water balance, tightly integrated with thirst.

## EVOLUTION OF ALDOSTERONE, SALT APPETITE, AND RENAL VULNERABILITY

In contrast to water balance, sodium balance underwent major changes with terrestrial life. In higher vertebrates, distal nephron segments are involved in sodium regulation through aldosterone, the main effector hormone of the RAAS [49, 50]. The mineralocorticoid receptor (MR) is already expressed in fish, but its specific mineralocorticoid ligand, aldosterone, appears only with terrestrial vertebrates, coinciding with the colonization of land around 400 million years ago [51–53]. Before that, MR was activated by cortisol or other corticosteroids [51].

Aldosterone exerts genomic actions through MR, regulating genes involved in sodium reabsorption and potassium excretion. Because glucocorticoids circulate at much higher concentrations, mineralocorticoid specificity required additional mechanisms, such as 11β-hydroxysteroid dehydrogenase-2, to protect MR from cortisol. The late emergence of aldosterone arguably provided more precise control of sodium balance and blood pressure to support higher physical and metabolic activity in terrestrial environments [49–53].

In humans, external sodium balance is largely controlled by aldosterone. When dietary sodium chloride is restricted from high to low intake, sodium balance is restored within days, but this requires pronounced hyperaldosteronism and genomic adaptation [54]. Aldosterone acts more slowly than ADH, whose effects are mediated by rapid cyclic adenosine monophosphate (cAMP) generation and aquaporin trafficking.

The threshold for hypovolaemic thirst is a loss of about 8%–10% of circulating volume, whereas osmotic thirst is triggered by a 1%–2% increase in plasma osmolality [45]. Unlike thirst, salt appetite seems a relatively weak and inconsistent mechanism for replacing sodium deficits. Even in conditions of aldosterone deficiency with volume depletion, such as Addison’s disease, clinically relevant salt craving is reported only in a minority of patients [55]. Neural circuits for salt hunger overlap with reward and addiction networks and lack a discrete ‘salt centre’ comparable to the thirst centre [33, 34].

These features suggest that the aldosterone-dependent system for sodium conservation is evolutionarily younger, slower, and behaviourally less compelling than the ADH–thirst axis. Under conditions of sustained sodium loss, such as prolonged sweating in hot environments, sodium balance may therefore be more fragile than water balance. In HSN, individuals may readily replace water but often fail to adequately replace sodium, predisposing to relative hypovolaemia and acute interstitial nephritis.

In parallel, terrestrial vertebrates evolved kidneys with high GFR and energy-dependent tubular reabsorption. While this strategy may be efficient for conserving water and solutes, it renders tubular cells, particularly in the outer medulla, highly sensitive to reductions in blood flow and oxygen delivery [36, 56–58]. Dehydration and hypovolaemia due to heat exposure may therefore destabilize intrarenal haemodynamics, activate tubulo-glomerular feedback and precipitate HSN, especially in older subjects and patients with CKD, hypertension, or diabetes, in whom microvascular and tubular resilience are already compromised [59, 60].

## CLINICAL IMPLICATIONS: INTEGRATING WATER, SODIUM/VOLUME, AND TEMPERATURE CONTROL

The clinical approach to HSN should mirror the three–way balance between thermoregulation, water homeostasis, and sodium-volume regulation.

First, reducing heat load and actively lowering core body temperature is fundamental. Priority should be given to environmental and behavioural interventions (work–rest cycles, access to shade, ventilation, appropriate clothing, and scheduling of the heaviest tasks during cooler hours), with active cooling strategies (cool water immersion and evaporative or conductive cooling) reserved for more severe heat stress or incipient heat illness.

Second, preserving effective circulating volume is essential to limit medullary hypoxia and recurrent ischaemic-inflammatory injury; this includes timely fluid replacement tailored to sweat losses and workload, alongside organizational measures that permit regular hydration breaks. Some additional hints for western wealthy countries, where older and obese are becoming the most common subgroups of general population, can be postulated. Indeed, elderly is characterized by a subtle and subclinical sodium loss [61] making older subjects prone to AKI even in the presence of normal blood pressure during hot periods that today are becoming very common and long [60]. Similarly, individuals with obesity are increasingly treated with drugs that reduce appetite; notably, a decrease of nutrients almost inevitably is associated with reduced salt intake, and again this can drive a reduction in eGFR during the hot season. Third, correction of hyperosmolality should proceed cautiously, with an emphasis on water replacement and judicious use of sodium–containing solutions. While combined water-salt rehydration may be appropriate for heavily sweating, otherwise healthy workers, it should be individualized and used with particular caution in people with hypertension, established CKD, heart failure, or high habitual sodium intake. In these vulnerable groups, aggressive sodium loading may exacerbate cardiovascular and renal risk, so strategies that prioritize cooling, adequate water intake, and volume preservation without routine sodium supplementation are preferable.

Overall, prevention and therapy should be individualized, context–specific, and explicitly acknowledge the trade–offs between protecting core temperature, sustaining effective volume, and avoiding harmful extremes of osmolar and sodium balance.

Finally, HSN should be viewed within the broader context of climate policy. Efforts to mitigate greenhouse gas emissions and adapt to rising temperatures could be relevant to kidney health [2–5]. Nephrologists, epidemiologists, and public health professionals should participate in interdisciplinary initiatives addressing the renal consequences of climate change, with a specific focus on HSN and heat-related CKD.

Several research needs follow directly. There is a need to refine clinical definitions and boundaries of HSN and to clarify how it relates to CKD of unknown aetiology, exertional heat-stroke-associated AKI, and other forms of kidney disease. Large, well-characterized cohorts with standardized definitions and longitudinal follow-up are required. The contribution of potential co-factors, such as environmental toxins, pesticides, infections, and genetic susceptibility, also remains incompletely understood [2, 3, 9, 10]. Integrated studies combining exposure assessment, biomarker profiling, and genetic or epigenetic analyses are needed.

## CONCLUSIONS

The impact of climate change on kidney health and the growing wave of HSN exemplify the clinical relevance of deeply rooted evolutionary features of renal physiology. The relative robustness of water regulation and the comparative weakness of sodium regulation, shaped over hundreds of millions of years, plausibly make the human kidney particularly vulnerable to modern heat stress. This vulnerability may, however, be mitigated by interventions that acknowledge and counteract our phylogenetic biases: proactive replacement of both water and sodium, protection of those most exposed to extreme heat, and early detection of heat-related kidney stress. Integrating contemporary clinical evidence with an evolutionary understanding of fluid and electrolyte homeostasis may provide a coherent framework for prevention and care of HSN in a warming world.

## Data Availability

The data underlying this article are available in the article.
